# Ileal hybrid schwannoma/perineurioma presenting with gastrointestinal bleeding: a case report

**DOI:** 10.3389/fsurg.2026.1778083

**Published:** 2026-04-23

**Authors:** Shiyu Pan, Changxi Chen, Gun Chen, Jiande Gong, Xianhao Ying, Hongliang Li

**Affiliations:** 1 Department of Gastroenterology, The Affiliated People's Hospital of Ningbo University, Zhejiang, China; 2 Department of Pathology, The Affiliated People's Hospital of Ningbo University, Zhejiang, China

**Keywords:** case report, gastrointestinal bleeding, hybrid schwannoma/perineurioma, ileal tumor, small bowel endoscopy

## Abstract

Hybrid schwannoma/perineurioma (HSP) is a rare benign peripheral nerve sheath tumor. Its occurrence in the gastrointestinal tract with associated bleeding is exceptionally uncommon. Definitive diagnosis relies critically on a complete surgical specimens and comprehensive immunohistochemical analysis. Complete surgical removal is the preferred treatment method and is associated with a favorable prognosis. We report the case of a 19-year-old male admitted with recurrent hematochezia without obvious cause. Small bowel endoscopy revealed an ulcerated, space-occupying lesion located approximately 120 cm from the ileocecal valve in the ileum. Initial biopsy was limited by a small tissue sample, rendering diagnosis challenging. Following laparoscopic resection of the small bowel lesion, pathological examination confirmed the diagnosis of ileal hybrid schwannoma/perineurioma. The patient recovered well postoperatively. Through this case, we aim to explore the clinicopathological characteristics, diagnostic approach, and therapeutic strategies for HSP, thereby enhancing awareness of this rare entity.

## Introduction

Hybrid schwannoma/perineurioma is a rare and morphologically distinct subtype of benign peripheral nerve sheath tumor (1, 2), characterized by dual differentiation toward Schwann cell and perineurial cell phenotypes (3). Although HSP is increasingly recognized in pathological practice, its clinical behavior, optimal management, and potential molecular mechanism remain incompletely understood. This report describes a case of ileal HSP presenting with gastrointestinal bleeding, aiming to improve recognition of this rare condition among clinicians and pathologists by detailing its clinicopathological features, diagnostic process, and treatment.

## Case presentation

A 19-year-old male presented with recurrent hematochezia for one week without obvious cause. He passed dark red bloody stools two to three times per day, approximately 100 mL each episode, accompanied by mild paroxysmal periumbilical abdominal pain. There was no nausea, vomiting, or cessation of flatus or defecation. Initial gastroscopy and colonoscopy performed at a local hospital revealed no definite bleeding source. Following symptomatic treatment with acid suppression, hemostasis, and fluid resuscitation, the hematochezia resolved and stool color normalized. However, due to persistent fecal occult blood positivity and suboptimal improvement in anemia, the patient was transferred to our department for further management. Physical examination showed stable vital signs and an anemic appearance, with no palpable abdominal mass, tenderness, or rebound tenderness. Laboratory findings revealed hemoglobin of 85 g/L, fecal occult blood 3+, and mildly decreased total serum protein at 54.8 g/L; The other test indicators showed no significant abnormalities.

**Figure 1:**
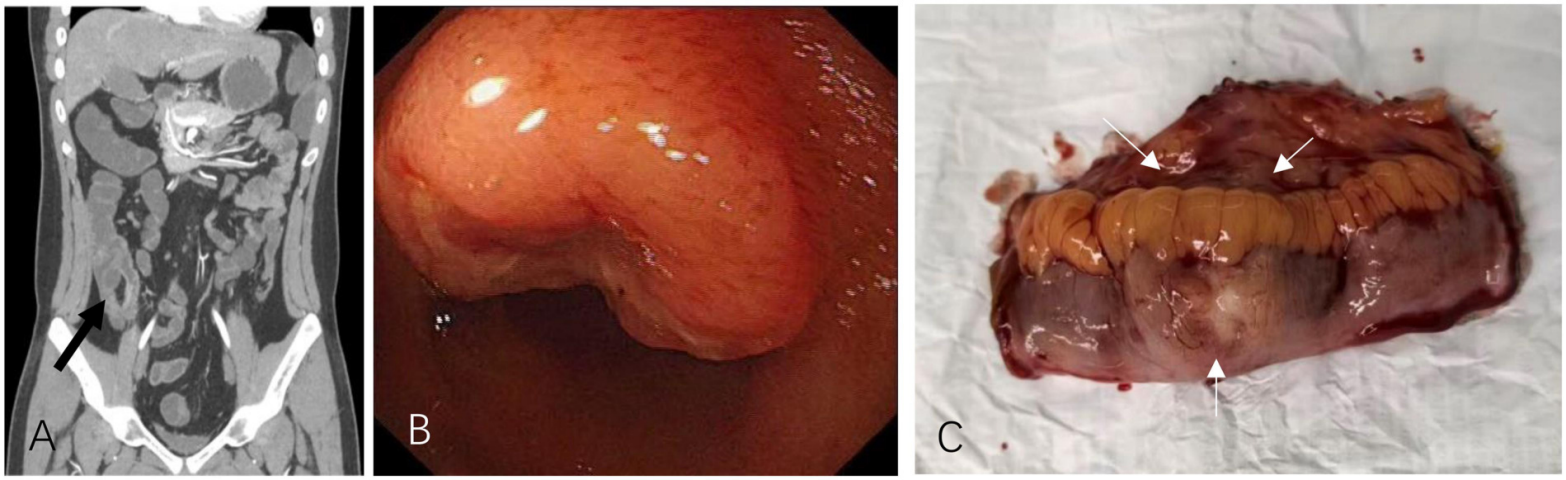
**(A)** the CT angiography (CTA) of the patient's small intestinal arteries are presented. As indicated by the arrow, there is eccentric thickening and enhancement at the lower end of the ileal intestinal wall, which suggests an inflammatory or vascular lesion. **(B)** Small bowel endoscopic view showing a ileal space-occupying lesion with surface ulceration; **(C)** The gross specimen obtained through surgery, shown in the picture is the outer surface of the small intestine. The arrow indicates the outline of the tumor.

To identify the source of small bowel bleeding, non-invasive computed tomography angiography (CTA) of the small intestine was performed, suggesting multiple hypervascular lesions in the ileum with associated inflammatory changes in the intestinal wall, consistent with inflammatory and vascular etiologies (Figure 1A). Subsequent capsule endoscopy revealed no bleeding site. Small bowel endoscopy was then performed, advancing to approximately 150 cm proximal to the ileocecal valve, followed by gradual withdrawal. A space-occupying lesion measuring approximately 2.0 cm was identified in the ileum, located about 120 cm from the ileocecal valve, occupying approximately half of the luminal circumference with central depression and ulceration (Figure 1B). Three biopsy specimens were obtained from the lesion, and a titanium clip was placed for localization. Initial pathological examination of the biopsy reported: “soft tissue tumor of vascular-rich origin with infiltrative growth within the lamina propria and submucosa, low proliferative activity, and surface ulceration.” Due to the limited tissue and atypical immunohistochemical expression, the initial consideration is a special type of hemangioblastoma or a gastrointestinal neurogenic tumor.

**Figure 2:**
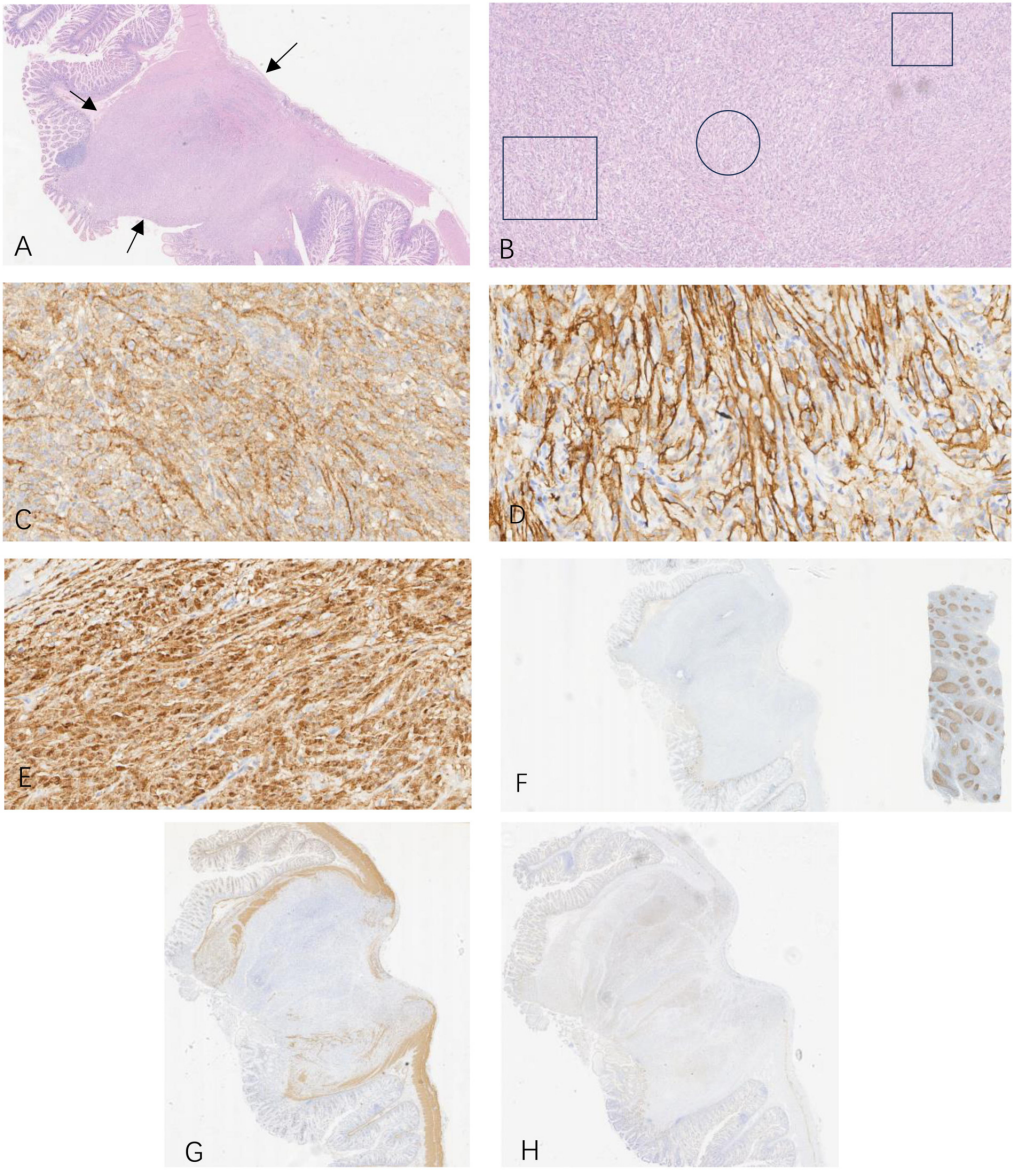
Pathological features of the hybrid schwannoma/perineurioma. **(A)**Low-power view of HE-stained tumor sections (arrows indicate the tumor outline); **(B)** Under a 4× magnification, the tumor cells show a distinct reticular distribution in the boxed area, which represents the morphological characteristics of a perineurioma. The circular area shows a loose reticular arrangement of cells, indicating the morphological features of schwannoma. Transitional migration can be observed between the two types of tumor cells; **(C–E)** Immunohistochemistry showing positive staining for EMA **(C)**, Glut-1 **(D)**, S100 **(E)**, and negative staining for Desmin **(G)** and DOG-1 **(H)**; **(F)** Ki-67 immunostaining showing an index of less than 5% within the tumor tissue (adjacent normal control tissue is shown on the right).

For definitive diagnosis and treatment, the patient was referred to the gastrointestinal surgery department for laparoscopic exploration. Intraoperatively, a firm mass measuring approximately 2.0 cm in diameter was found in the ileum, approximately 1.2 m proximal to the ileocecal valve, without adhesions to surrounding tissues. Segmental resection of the small bowel tumor was performed. Gross examination of the resected specimen revealed a grayish-white, well-demarcated mass within the intestinal wall (Figure 1C). Microscopic examination showed tumor cells infiltrating the full thickness of the intestinal wall. In some areas, the cells were densely arranged in palisading patterns, while in other areas, they were sparsely distributed within collagen bundles. Immunohistochemical staining revealed diffuse, strong positivity for S-100 and SOX-10 (consistent with the immunohistochemical expression of schwannoma) as well as positive staining for EMA and Glut-1 (consistent with the immunohistochemical expression of perineurioma). DOG-1 and Desmin were negative. The Ki-67 proliferative index was approximately 5% (Figure 2). Integrating the immunohistochemical and morphological features, the final pathological diagnosis was “hybrid schwannoma/perineurioma”. The report noted focal areas of increased cellular activity with low-grade malignant potential. Resection margins were negative.

On postoperative day 9, abdominal CT showed “postoperative changes after small bowel resection, multiple mesenteric lymph nodes in the mid-to-lower abdomen, and widening of the left inguinal canal.” During this period, gastrointestinal bleeding ceased, hemoglobin levels recovered, and fecal occult blood testing became negative. The patient was discharged successfully.

## Discussion

In our case, we observed a close admixture of S-100/SOX-10-positive Schwann cell areas (often associated with Verocay bodies or hyalinized vessels) (4) and EMA-positive perineurial cell areas (exhibiting storiform or whorled patterns). This dual immunophenotype is not merely an intriguing pathological finding but is critical for accurate classification and for distinguishing HSP from its histological mimics. For instance, conventional schwannomas show uniform strong positivity for S-100, whereas conventional perineuriomas are diffusely positive for EMA and negative for S-100. In this case, the two components were intimately interwoven microscopically.

Reports of hybrid schwannoma/perineurioma arising in the gastrointestinal tract are rare, and ileal localization is exceptionally uncommon. While one prior case described an esophageal schwannoma diagnosed in a patient with chronic iron deficiency anemia refractory to supplementation, no direct causal relationship between the anemia and the tumor was established in that report (5). In contrast, our patient presented with overt gastrointestinal bleeding, and the relationship between the bleeding and the tumor was clearly demonstrated by endoscopic findings and postoperative outcomes.

Complete surgical excision is the preferred and effective treatment (6). In this case, segmental small bowel resection with negative margins achieved curative intent. The vast majority of HSPs are benign, with minimal risk of recurrence after complete resection. However, a small number of cases with atypical features or low malignant potential have been reported. Malignant peripheral nerve sheath tumors typically exhibit marked cytologic atypia, frequent mitotic figures, necrosis, and often a higher Ki-67 index. Although our case showed focal “increased cellularity,” the Ki-67 index was low (5%), and no definitive malignant features were identified, suggesting benign or low-grade malignant potential (7), warranting long-term follow-up.

Although this patient presented with a solitary lesion and no clear family history, the occurrence of HSP is closely associated with schwannomatosis. Schwannomatosis is primarily caused by germline mutations in the SMARCB1 or LZTR1 genes and is characterized by multiple schwannomas or HSP (8, 9). Therefore, thorough clinical evaluation such as whole-body imaging and genetic counseling are essential for all HSP patients, particularly young individuals, to rule out underlying genetic syndromes and to guide screening of family members.

## Conclusion

This case report provides the first detailed description of ileal hybrid schwannoma/perineurioma presenting with gastrointestinal bleeding. It highlights that rare neurogenic tumors should be considered in the differential diagnosis of obscure gastrointestinal bleeding, particularly of small bowel origin. Accurate diagnosis depends on recognition of the tumor's distinctive histological features by pathologists and the use of a comprehensive application of immunohistochemical markers. Surgical resection yields favorable outcomes, but postoperative screening for associated genetic syndromes and long-term follow-up are important.

## Data Availability

The original contributions presented in the study are included in the article/Supplementary Material, further inquiries can be directed to the corresponding author.
